# The Immunomodulatory Functions of Diacylglycerol Kinase ζ

**DOI:** 10.3389/fcell.2016.00096

**Published:** 2016-09-07

**Authors:** Brenal K. Singh, Taku Kambayashi

**Affiliations:** Department of Pathology and Laboratory Medicine, Perelman School of Medicine at the University of PennsylvaniaPhiladelphia, PA, USA

**Keywords:** diacylglycerol, diacylglycerol kinase, signal transduction, immunomodulation, phospholipase C, extracellular signal-regulated kinase, phosphatidic acid

## Abstract

The generation of diacylglycerol (DAG) is critical for promoting immune cell activation, regulation, and function. Diacylglycerol kinase ζ (DGKζ) serves as an important negative regulator of DAG by enzymatically converting DAG into phosphatidic acid (PA) to shut down DAG-mediated signaling. Consequently, the loss of DGKζ increases DAG levels and the duration of DAG-mediated signaling. However, while the enhancement of DAG signaling is thought to augment immune cell function, the loss of DGKζ can result in both immunoactivation and immunomodulation depending on the cell type and function. In this review, we discuss how different immune cell functions can be selectively modulated by DGKζ. Furthermore, we consider how targeting DGKζ can be potentially beneficial for the resolution of human diseases by either promoting immune responses important for protection against infection or cancer or dampening immune responses in immunopathologic conditions such as allergy and septic shock.

## Introduction

Diacylglycerol (DAG) is a key secondary lipid messenger for transducing signals downstream of many receptors expressed by hematopoietic cells. DAG has shown to be important in driving the activation, proliferation, migration, and effector function of adaptive and innate immune cells. The generation of DAG can be accomplished by the activation of various cell-surface receptors, including G_q_-mediated G-protein coupled receptors (GPCR)s (e.g., muscarinic and histamine receptors) and immunoreceptor tyrosine-based activation motif (ITAM)-bearing receptors [e.g., T cell receptor (TCR), FcεRI] (Topham and Prescott, 1999; Kambayashi and Koretzky, 2007; Smith-Garvin et al., 2009; Wright et al., 2013). The activation of these receptors results in the formation of proximal signaling complexes that are critical for the activation of phospholipase C (PLC). PLC activation leads to enzymatic cleavage of phosphoinositol 4,5-bisphosphate (PIP_2_) into DAG and inositol 1,4,5-triphosphate (IP_3_) (Imboden and Stobo, 1985). While IP_3_ mobilizes Ca^2+^, DAG activates the NF-κB and extracellular regulated kinase (ERK) pathways through protein kinase C (PKC) and RasGRP, respectively, to promote cell function (Tognon et al., 1998; Coudronniere et al., 2000; Dower et al., 2000; Sun et al., 2000; Wang et al., 2004; Quann et al., 2011). Consequently, the levels of DAG must be tightly regulated to control the magnitude and duration of the responses generated.

Diacylglycerol kinases (DGK) regulate DAG signaling by phosphorylating DAG and converting it into phosphatidic acid (PA) (Topham and Prescott, 1999; Joshi and Koretzky, 2013). The loss of DGKs increases DAG levels and the duration of DAG-mediated signaling. One might expect that elevated DAG levels would lead to general enhancement of effector responses. However, the enhancement of DAG signaling through the loss of DGKs can lead to either hyperactivation or hyporesponsiveness depending on the cell type and the type of response. There are 10 different isoforms comprising five different classes of DGKs, each of which control different cellular functions based on their distinct structural motifs and subcellular localization (Bunting et al., 1996; Goto and Kondo, 1996; Topham and Prescott, 1999; Kobayashi et al., 2007; Joshi and Koretzky, 2013). The three major isoforms that are abundantly expressed in lymphoid tissues are DGKα, DGKδ, and DGKζ (Shulga et al., 2011). In particular, mice that lack the zeta (ζ) isoform of DGK, which is highly expressed in hematopoietic cells, display profound effects on the functional behavior of various cell types. In the present review, we will focus on how DGKζ plays both negative and positive roles in immune responses mounted by different cell types.

## Negative regulation of effector responses by DGKζ

### CD4^+^ and CD8^+^ conventional T cells

DGKζ serves as a critical negative regulator of DAG signaling downstream of the TCR and can modulate the strength of TCR signaling. Early experiments using the immortalized Jurkat T cell line showed that overexpression of DGKζ inhibits TCR signaling by reducing the levels of active GTP-bound Ras and, consequently, diminishing ERK activation (Zhong et al., 2002). Furthermore, the overexpression of DGKζ was associated with decreased AP-1 transcription factor activity and CD69 expression (an early T cell activation marker) following TCR stimulation, both of which are regulated by the Ras-ERK pathway. Importantly, Ca^2+^ flux was normal regardless of DGKζ overexpression, suggesting that DGKζ selectively regulated DAG-mediated signaling pathways downstream of the TCR. Further biochemical analysis through the use of a kinase dead DGKζ mutant revealed that the enzymatic activity of DGKζ was critical for its inhibitory effects on TCR signaling.

To test the physiological role of DGKζ in T cells, Zhong et al. generated DGKζ knockout mice (Zhong et al., 2003). Initial phenotypic analysis showed that DGKζ KO mice contained similar frequencies and numbers of CD4^+^ and CD8^+^ T cells in secondary lymphoid organs and displayed no obvious defects in lymphoid architecture or cellularity. Furthermore, thymic development as analyzed by the number and frequency of CD4 single-positive (SP), CD8 SP, double-positive (DP), and double-negative (DN) thymocytes in DGKζ-deficient mice was similar to wild-type (WT) mice. However, upon TCR stimulation, naïve DGKζ KO CD4^+^ and CD8^+^ T cells displayed enhanced upregulation of activation markers CD25 and CD69 and increased proliferation compared to WT T cells. The increased expression of activation markers was associated with enhanced phosphorylation of ERK but normal induction of non-DAG mediated pathways including Ca^2+^ flux and JNK activation. Importantly, bypassing TCR activation with a DAG analog, phorbol-12-myristate-13-acetate (PMA), abolished differences in activation between DGKζ KO and WT T cells, suggesting that the hyperactivation of DGKζ KO T cells was secondary to defective regulation of DAG.

In agreement with enhanced TCR signaling, DGKζ KO T cells also display improved anti-viral responses (Zhong et al., 2003). DGKζ KO mice infected with LCMV Armstrong showed enhanced viral-specific T cell responses as evidenced by decreased viral titers at day 7 following infection. This effect correlated with an increased number of total and CD44^hi^CD62L^lo^ effector CD8^+^ T cells in the spleen. Furthermore, LCMV-infected DGKζ KO mice exhibited increased number of IFNγ-producing CD8^+^ and CD4^+^ T cells when restimulated with LCMV-specific peptides, suggesting that DGKζ KO T cells displayed enhanced effector function following LCMV infection.

Similarly, DGKζ-deficient mice also exhibit enhanced anti-tumor responses. DGKζ KO mice subcutaneously injected with OVA-expressing EL4 T cell lymphoma, had significantly reduced tumor mass compared to their WT counterparts (Riese et al., 2011). This effect was accompanied by an increased number of total and antigen-specific tumor-infiltrating CD44^hi^CD8^+^ T cells. To show that T cells were responsible for the enhanced anti-tumor effect by DGKζ deficiency, DGKζ KO and WT OVA-specific OT-I T cells were adoptively transferred into naïve recipient WT mice. Mice receiving DGKζ KO compared to WT OVA-specific OT-I T cells also exhibited lower tumor mass upon challenge with OVA-expressing EL4 cells. Isolation of tumor-infiltrating OT-I cells revealed that the loss of DGKζ increased the frequency of CD44^hi^ and IL-2-producing OT-I cells in a cell-intrinsic manner. In addition to preventing tumor engraftment, DGKζ deficiency also improves tumor rejection of established tumors, as the adoptive transfer of DGKζ KO but not WT OT-I effector T cells into tumor-bearing mice significantly reduced tumor burden (Riese et al., 2013). Thus, DGKζ could represent a novel target for enhancing anti-tumor responses in adoptive immunotherapy. This could also be applied to engineered T cells that express chimeric antigen receptors (CAR) directed against the tumor, as DGKζ deficiency was also shown to promote CAR T cell-mediated anti-tumor responses (Riese et al., 2013). How DGKζ deficiency augments anti-tumor responses is unclear. Although DGKζ KO CD8^+^ T cells display increased cytokine production and increased proliferation, their cytotoxic function is comparable to WT CD8^+^ T cells (Riese et al., 2011). Nevertheless, these studies demonstrate that DGKζ serves to constrain T cell activation and anti-viral and anti-tumor T cell responses. Thus, inhibition of DGKζ might provide a therapeutic opportunity to enhance immune-mediated viral and tumor clearance.

It is possible that DGKζ is physiologically important for limiting over-activation and inducing anergy in inappropriately activated T cells. The expression level of DGKζ can be controlled depending on the type of stimulation the T cell receives. T cells that are stimulated through their TCR and co-stimulatory molecules downregulate DGKζ transcript levels, thereby allowing appropriately activated T cells to become fully activated (Macian et al., 2002; Olenchock et al., 2006a; Zha et al., 2006). In contrast, T cells that receive TCR stimulation alone in the absence of co-stimulation do not downregulate DGKζ levels, potentially leading to attenuated DAG-mediated signaling and decreased activation. Consistent with this notion, DGKζ KO T cells resist anergy induction when activated by TCR alone in the absence of co-stimulatory signals (Olenchock et al., 2006a). In addition to TCR-mediated regulation, DGKζ might also be regulated by environmental cytokines. In particular, IL-33 has been shown to up-regulate DGKζ in cardiomyocytes following stimulation (Rui et al., 2012). Although it is unknown if IL-33 can upregulate DGKζ in immune cells, it is tantalizing to speculate that cytokine signaling can affect the TCR responsiveness of T cells by regulating DGKζ levels.

### NK cells

NK cells are cytotoxic members of the innate lymphoid cell (ILC) family and play an important role in protection against viral infection and clearance of tumors (Artis and Spits, 2015). Unlike their adaptive counterparts (CD8^+^ T cells), they do not possess a somatically-rearranged antigen receptor but rather express a variety of activating receptors specific for ligands displayed on virally-infected, stressed, or transformed cells (Lanier, 2008). NK cell activating receptors can be categorized into three main families based on the signaling adaptors used to relay downstream activation signals. These families include SAP-dependent (e.g., 2B4), ITAM-dependent (e.g., CD16), or DAP10-dependent (e.g., NKG2D) receptors (McVicar et al., 1998; Wu et al., 1999; Chen et al., 2006; Lanier, 2008). The activation of any of these three families of receptors relies on proximal signaling complexes involving SLP-76, which subsequently leads to the activation of PLCγ and the production of DAG (Wu and Koretzky, 2004; Tassi et al., 2005; May et al., 2013). In addition to these activating receptors, NK cells express an assortment of inhibitory receptors, many of which bind to MHC class I alleles and negatively regulate activating receptor signaling by the recruitment of phosphatases such as SHP-1 and SHIP (Binstadt et al., 1996; Lanier, 2008).

NK cell activation is determined by the net balance of the activating and inhibitory inputs that the NK cell receives through its receptors. For example, NK cells are activated when neoplastic cells upregulate ligands such as RAE-1 or MICA, which are recognized by the activating receptor NKG2D (Jung et al., 2012). Likewise, NK cells are activated through disinhibition when tumor cells lose MHC class I, a process known as missing self recognition (Kärre et al., 1986). Since SHP-1 and SHIP negatively regulate activating receptor signaling, one might predict that the loss of these molecules would boost the effector function of NK cells. Surprisingly, however, SHP-1 and SHIP deficiency in NK cells renders them less functional than their WT counterparts (Lowin-Kropf et al., 2000; Viant et al., 2014; Gumbleton et al., 2015). One explanation of this seemingly paradoxical finding is that NK cells continuously adjust their responsiveness to activating stimuli in their local environment, a phenomenon known as tuning (Höglund and Brodin, 2010). Thus, NK cells that chronically lack inhibitory signals, such as in SHP-1 or SHIP deficiency, require more stimulation to achieve their threshold of activation (Lowin-Kropf et al., 2000; Viant et al., 2014; Gumbleton et al., 2015). While NK cell tuning may protect the host from NK cell-mediated immunopathology, this process can hamper important effector responses against chronic viral infections or tumors.

Although the molecular mechanism of NK cell tuning is unknown, stimulation of NK cells with PMA and a calcium ionophore, ionomycin, can bypass the hyporesponsiveness of SHP-1 and SHIP KO NK cells (Viant et al., 2014; Gumbleton et al., 2015). These data suggest that the tuning process is proximal to PLCγ-mediated production of DAG. Thus, we speculated that NK cells may not be able to tune their responsiveness in response to enhanced DAG-mediated signaling by DGKζ deficiency. Indeed, we recently demonstrated that DGKζ KO NK cells are hyperfunctional compared to WT NK cells (Yang et al., 2016). DGKζ KO NK cells displayed increased cytokine production and cytotoxicity following stimulation through ITAM, SAP, and DAP10-dependent activating receptors. In contrast, IFNγ production by DGKζ KO and WT NK cells was similar following stimulation with IL-12 and IL-18, which utilize a DAG-independent signaling pathway, suggesting that the loss of DGKζ selectively augmented NK cell responsiveness to DAG-dependent stimuli. Like T cells, the hyperfunctionality of DGKζ KO NK cells was dependent on enhanced ERK signaling. Importantly, DGKζ KO mice cleared the NK cell-sensitive RMA-S tumor more efficiently than WT mice. Thus, the inactivation of negative regulators distal to PLCγ such as DGKζ might prove therapeutically useful in enhancing NK cell function.

### B cells

B cells comprise the second arm of the adaptive immune system and are critical for the generation of protective antibody responses during infection. The induction of antibody production results from the stimulation of the somatically rearranged B cell receptor (BCR) by cognate antigen (McHeyzer-Williams and McHeyzer-Williams, 2005; Kurosaki et al., 2010). Similar to the TCR, activation of the BCR leads to downstream biochemical cascades that ultimately result in the generation of DAG through PLCγ and, subsequently, the activation of ERK (Hashimoto et al., 2000; Saijo et al., 2003). ERK has been shown to play multiple roles during B cell responses, including the promotion of B cell survival, proliferation, and differentiation into antibody-secreting plasma cells (Richards et al., 2001; Coughlin et al., 2005; Yasuda et al., 2011). Furthermore, attenuation of ERK activation has been shown to important during B cell development, since ERK signals decrease as B cells progress from the immature transitional stage to mature follicular B cells (Yasuda et al., 2008; Gross et al., 2009; Rowland et al., 2010).

Given the role of ERK in these B cell processes, controlling the levels of BCR-induced DAG through DGKζ might be important in regulating B cell development, activation, and antibody secretion capabilities. For example, mRNA transcripts of DGKζ are upregulated as B cells progress from early transitional to the mature follicular stage, which is associated with decreased ERK activation (Wheeler et al., 2013). Accordingly, the loss of DGKζ only affected ERK activation and IκBα degradation in the follicular but not early immature transitional B cell pool in response to BCR stimulation. Importantly, the augmentation of BCR-induced activation in DGKζ KO follicular B cells was seen even under less optimal BCR activation conditions, suggesting that DGKζ might control the BCR activation threshold in these cells.

The effects of DGKζ on B cell signaling threshold translate to functional consequences on B cell effector responses. BCR stimulation of purified DGKζ KO splenic B cells *in vitro* led to increased expression of CD69 and enhanced proliferation compared to WT B cells. DGKζ KO mice displayed enhanced antibody responses to T-independent and T-dependent antigens (Wheeler et al., 2013). The heightened antibody response by DGKζ-deficiency was accompanied by increased antigen-specific expansion of both germinal center (GC) B cells and plasma cells. These results demonstrate that regulation of DAG-dependent ERK activation by DGKζ is critical for selectively controlling the activation threshold of mature B cells to limit their activation.

## The immunomodulatory role of DGKζ

We have so far described how the loss or inhibition of DGKζ can lead to increased immune responses against viruses or cancer. As DGKζ is a negative regulator of DAG-mediated signaling, it is conceivable that immune responses would be enhanced in the absence of DGKζ. However, DGKζ deficiency may also lead to dampening or regulation of immune responses. In the sections below, we will discuss how the absence of DGKζ can direct and indirectly suppress or modulate rather than enhance immune responses.

### Regulatory T cells

Regulatory T cells (Tregs) are a key subset of T cells that display suppressive function and are important for the regulation of adaptive immune responses. Tregs are governed by the master transcription factor, forkhead box P3 (Foxp3), and exert their immunosuppressive function via the production of immunoregulatory cytokines and through cell contact dependent mechanisms (Josefowicz et al., 2012). Loss of function mutations in the *Foxp3* gene, as seen in Scurfy mice and humans with immune dysregulation, polyendocrinopathy, and X-linked lymphoproliferative disease (IPEX), leads to lethal systemic autoimmunity early in life, highlighting the importance of Tregs in inducing immunotolerance against self antigens (Chatila et al., 2000; Bennett et al., 2001; Brunkow et al., 2001; Wildin et al., 2001).

T cells that strongly recognize self antigens are deleted during thymic development in a process known as negative selection. Specifically, T cells that receive strong TCR signals in the thymus, implying overt self reactivity, undergo apoptosis. As an alternative fate, strong TCR stimulation in developing thymocytes can also lead to Treg differentiation (Josefowicz et al., 2012). Thus, we hypothesized that enhancement of TCR-mediated DAG signaling by DGKζ deficiency in developing thymocytes may increase Treg generation. Indeed, the loss of DGKζ resulted in a significant increase in Treg development in the thymus in a cell-intrinsic manner (Schmidt et al., 2013). DAG-mediated signaling leads to the activation of the NF-κB (through activation of PKC) and ERK pathways. One NF-κB family member, c-Rel, was previously shown to be important for inducing Foxp3 expression in thymocytes (Long et al., 2009; Ruan et al., 2009). Although Treg generation in DGKζ KO mice was reduced in the absence of c-Rel, there was still residual Tregs in the thymus, suggesting that c-Rel was only partially responsible for the increased generation of Tregs in DGKζ KO mice (Schmidt et al., 2013). In fact, ERK activation appeared to be more important in the enhancement of Treg generation in DGKζ KO mice. Using an *in vitro* Treg development assay, we found that the inhibition of ERK phosphorylation by a MEK inhibitor led to decreased Treg generation in a dose-dependent manner, whereby the level of phosphorylated ERK (pERK) directly correlated to the magnitude of Treg generation. Importantly, Treg generation was also increased in sevenmaker mice (Sharp et al., 1997), which express a gain of function ERK mutation that leads to increased resistance to dephosphorylation of active pERK, suggesting that the selective enhancement of the ERK pathway alone is sufficient to increase Treg generation.

In addition to Treg generation in the thymus, TCR signaling plays an important role in the function of Tregs. Although some Treg function may be preserved in the absence of TCR signaling, we demonstrated that Tregs lacking SLP-76 cannot suppress TCR-driven proliferation of conventional T cells (Schmidt et al., 2015). Furthermore, Tregs with a Y → F mutation at tyrosine 145 (Y145F) of SLP-76, which leads to defective PLCγ activation, also display attenuated suppressive function, suggesting that PLCγ is important for Treg function. Consistent with this notion, Tregs lacking DGKζ display significantly increased suppression of TCR-driven conventional T cell proliferation compared to WT Tregs. Together, these data demonstrate that DGKζ limit Treg generation and function. Thus, DGKζ deficiency may indirectly lead to the suppression of immune responses through Tregs.

### Mast cells

Mast cells are critical mediators in type 2 immune responses involved in protection against helminthes and in pathologic responses in asthma and allergy (Locksley, 2010; Voehringer, 2013). A key feature of mast cell function is the immediate release of pre-formed inflammatory mediators such as histamine, cytokines, and proteases in a process called degranulation. In addition, mast cells produce arachidonic acid metabolites and cytokines in a protracted manner (Voehringer, 2013). One major stimulus for the release of these inflammatory mediators is crosslinking of FcεRI, the high affinity receptor for the Fc region of immunoglobulin E (Kinet, 1999).

The interaction of allergens with IgE-FcεRI complexes results in formation of signaling complexes that converge on the activation of PLCγ (Atkinson et al., 1992; Schneider et al., 1992). PLCγ and subsequent PKC activation have been shown to be critical in controlling mast cell degranulation, suggesting that controlling the levels of DAG might be important for regulating this process (Nechushtan et al., 2000; Wang et al., 2000; Leitges et al., 2002; Wen et al., 2002). Indeed, the loss of DGKζ in FcεRI-stimulated mast cells leads to increased DAG levels, along with enhancement of downstream DAG-dependent signals, including RasGTP and ERK (Olenchock et al., 2006b). Accordingly, DGKζ deficiency leads to enhanced mast cell production of IL-6 following FcεRI stimulation.

Intriguingly, however, FcεRI-stimulated DGKζ KO mast cells display impaired degranulation and are resistant to local skin anaphylaxis (Olenchock et al., 2006b). The differential effect of DGKζ deficiency on mast function (the hypersecretion of IL-6 vs. decreased degranulation) may be explained by the negative feedback of DAG on PLCγ activation in mast cells. The elevation of DAG by DGKζ deficiency appears to negatively regulate the phosphorylation and subsequent activity of PLCγ. Thus, although DAG accumulates, the production of IP_3_, and hence Ca^2+^ flux is attenuated in DGKζ KO mast cells. As degranulation responses are highly dependent on elevation of intracellular Ca^2+^ levels, this may cause a differential effect on degranulation and cytokine production by mast cells (Ozawa et al., 1993; Olenchock et al., 2006b). Thus, as opposed to T cells and NK cells, DGKζ exerts both activating and inhibitory effects on mast cell functional responses.

### Macrophages and dendritic cells

Macrophages and dendritic cells (DC) play a key role in bridging the adaptive and innate immune responses (Medzhitov, 2001; Janeway and Medzhitov, 2002; Akira and Takeda, 2004). Toll-like receptors (TLR) serve as an important mechanism for equipping macrophages and DCs with the ability to recognize the presence of pathogenic infection and, subsequently, instruct adaptive immune cells on the type of response needed to effectively clear the infection. TLRs can signal through either MyD88 and/or TRIF to induce activation of the NF-κB and ERK pathways (Akira and Takeda, 2004). While TLR activation does not generally lead to PLCγ activation, DAG has been shown to be induced in macrophages following stimulation with LPS (TLR4 agonist) and lipopeptide (TLR2 agonist) (Monick et al., 1999; Zhang et al., 2001a,b). Furthermore, inhibition of PLC or PLD reduced cytokine production and nitric oxide formation by macrophages following TLR stimulation, suggesting that control of DAG levels through DGK might be important in regulating TLR-mediated responses.

Interestingly, modulation of DAG levels by the loss of DGKζ resulted in impairment rather than enhancement of cytokine production by macrophages and DCs in response to TLR stimulation. Specifically, in a developmentally independent manner, bone marrow derived macrophages (BMMΦ) and splenic DCs produced substantially less IL-12p40 and TNFα following *in vitro* stimulation through a variety of TLR agonists (Liu et al., 2007). This paradoxical finding may be explained by the role of DGK in converting DAG into PA. Biochemical analysis revealed that the loss of DGKζ resulted in selective elevation of the PI3K-Akt pathway but no difference in activation of the ERK or NK-κB pathways following TLR stimulation. Activation of the PI3K pathway has been shown to negatively regulate TLR stimulation (Fukao et al., 2002; Guha and Mackman, 2002; Martin et al., 2005) and chemical inhibition of the PI3K restored LPS-induced IL-12p40 production from DGKζ KO BMMΦs (Liu et al., 2007). Intriguingly, the addition of PA also restored LPS-induced IL-12p40 production, suggesting that the cytokine production defect in DGKζ KO DCs and macrophages may be due to reduced PA rather than elevated DAG levels. Exactly how PA rescues TLR-induced cytokine production is unknown, but one possible mechanism is through the recruitment of SHP-1 to negatively regulate PI3K activation (Cuevas et al., 1999; Frank et al., 1999; Zhang et al., 2003).

Defective cytokine production was also observed *in vivo* following intraperitoneal injection of TLR agonists, which correlated with enhanced survival of DGKζ KO mice after LPS-induced septic shock (Liu et al., 2007). However, while DGKζ KO mice were protected from TLR-mediated pathology, the loss of DGKζ conferred susceptibility to *Toxoplasma gondii*. DGKζ KO mice infected with *T. gondii* displayed decreased serum IL-12p40 and IFNγ levels compared to WT mice. Furthermore, IFNγ production by DGKζ KO splenocytes isolated at day 15 and 30 post-infection was significantly attenuated following restimulation with *T. gondii* antigen STAg. Intriguingly, total CD4^+^ and CD8^+^ T cell numbers were similar between WT and DGKζ KO mice following infection with the frequency of CD44^+^CD62^lo^ effector T cells higher in infected DGKζ KO mice. As TLR-induced IL-12p40 production and the subsequent induction of a Th1 response are critical for protection against *T. gondii* infection, the impairment of immune responses against *T. gondii* by DGKζ KO mice could be secondary to a defect in macrophage and DC-derived cytokines that drive Th1 responses.

## The role of other DGK isoforms on DGKζ-regulated immune function

So far, we have discussed isoform-specific regulation of immune function by DGKζ, however it is possible that the loss of DGKζ has other functional consequences that might be masked by redundant functions of other DGK isoforms. Indeed, DGKα has been shown to display some redundant function with DGKζ during conventional T and invariant NKT cell (iNKT) development. While singly-deficient DGKα KO and DGKζ KO mice display no gross defects in thymic T cell development, mice deficient in both DGKα and DGKζ (DGKαζ DKO) have significant reductions in CD4 and CD8 SP populations in the thymus due to a cell-intrinsic block in positive selection from the DP to SP stage (Guo et al., 2008). Interestingly, the addition of PA to fetal thymic organ cultures could partially restore T cell maturation defect in DGKαζ DKO thymocytes, suggesting that DGKα and DGKζ regulate T cell development partly through redundant production of PA.

Similarly, the development of iNKT cells is intact in mice singly-deficient for either DGKα or DGKζ (Shen et al., 2011). However, the loss of both DGKα and DGKζ results in a complete impairment of iNKT cell maturation in the thymus, spleen, and liver at both early and terminal stages in a cell-intrinsic manner. Selective enhancement of the ERK pathway through the expression of a constitutively active K-ras resulted in a significant reduction in mature iNKT cells due a block in Stage II to Stage III maturation of iNKT precursors. Furthermore, augmented activation of the NF-κB pathway through the expression of a constitutively active IKKβ also resulted in an impairment in iNKT maturation at both early and terminal stages of development. These results suggest that DGKα and DGKζ play redundant roles in the regulation of iNKT maturation by controlling DAG-mediated activation of the ERK and NF-κB pathways.

In addition to controlling innate and conventional T cell development, DGKα has also been shown to promote T cell anergy in conjunction with DGKζ. Overexpression of either DGKα or DGKζ in Jurkat T cells induces an anergic-like state that is highlighted by reduced DAG-dependent TCR signals without the impairment of calcium flux (Olenchock et al., 2006a). Similar to DGKζ KO T cells, T cells deficient in DGKα resist anergy induction when activated through their TCR in the absence of costimulation and during superantigen-induced activation. Furthermore, pharmacological inhibition of DGKα in DGKζ-deficient T cells can further enhance proliferation and IL-2 production in response to anergy-inducing conditions, suggesting that both DGKα and DGKζ contribute to anergy induction in inappropriately activated T cells through the synergistic regulation of TCR-induced DAG-mediated signaling.

While DGKα and DGKζ can share similar functions, DGKα does not simply compensate for all DGKζ-regulated functions. For example, unlike DGKζ KO mice, DGKα-deficient mice do not display an enhancement in Treg generation in the thymus or hyperfunctional NK cell responses, thus emphasizing that the regulation of these processes by DGKs is isoform-specific and unique to DGKζ (Joshi et al., 2013; Yang et al., 2016). The independent and redundant roles of DGKα and other DGK isoforms on DGKζ-regulated functions in other immune cells remain unexplored.

## Concluding remarks

**Figure 1:**
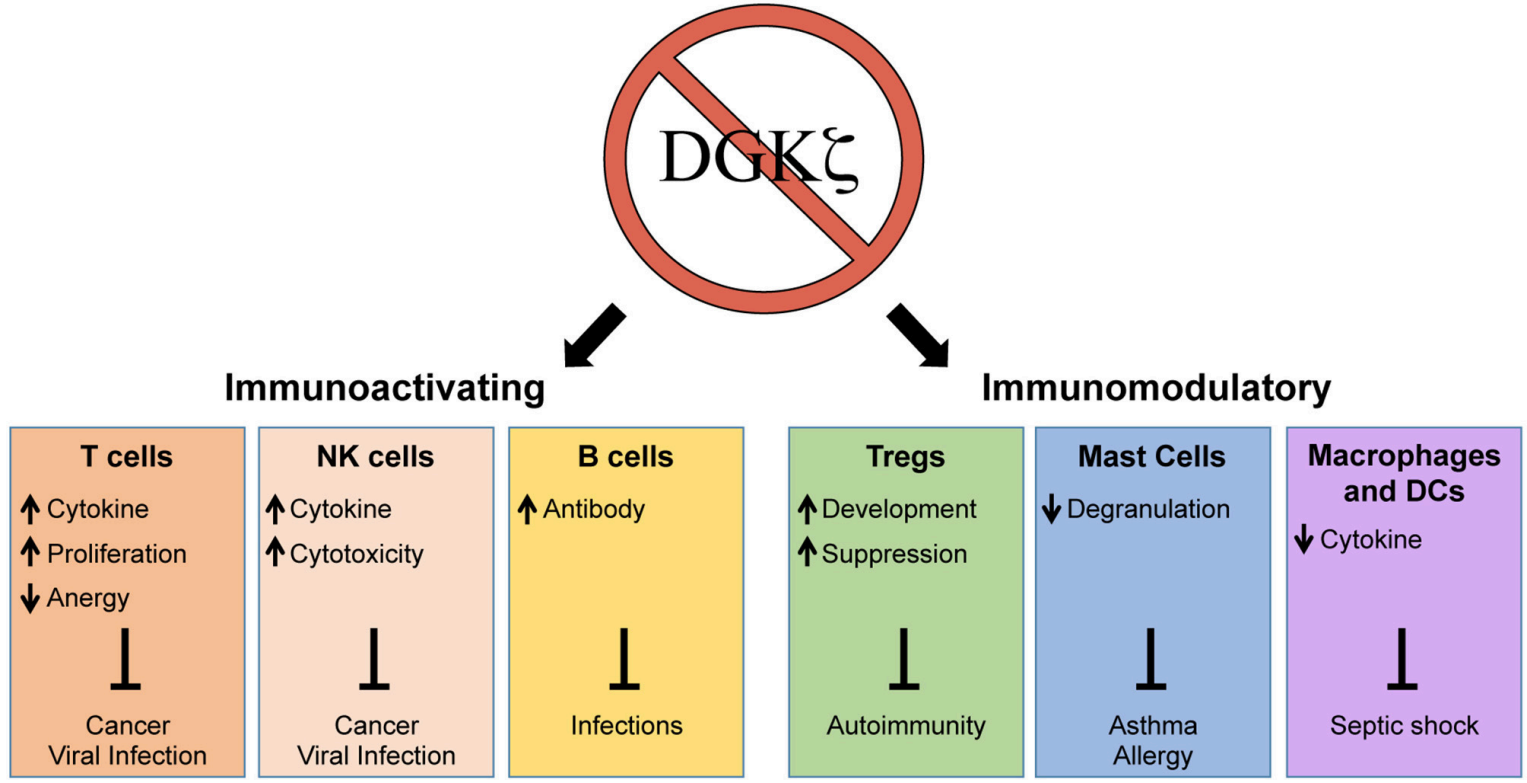
**Enhancement and suppression of selective immune functions through the inhibition of DGKζ**. The loss of DGKζ can result in activating or modulatory effects on the immune response that can be therapeutically beneficial for the resolution or prevention of a variety of human diseases. The inhibition of DGKζ has positive effects on T cells, NK cells, and B cells, which could promote immunity against cancer and infections. In contrast, the inhibition of DGKζ can suppress immune responses by augmenting Treg development, inhibiting mast cell degranulation, and attenuating macrophage/DC cytokine release. These effects could be beneficial in treatment of autoimmunity, asthma/allergy, and septic shock.

As a negative regulator of DAG-mediated signaling, one might predict that the loss of DGKζ would universally lead to immune activation. Interestingly, however, the inhibition of DGKζ does not only enhance but also suppresses selective immune responses (Figure 1). Thus, although DGKζ may represent a drug target for enhancing cytotoxic T cell responses against cancer, it may also simultaneously serve as a target for the treatment of allergic responses or septic shock. In addition to activation or inhibition of immune responses, DGKζ may also play a role in modulating the immune response. For example, it has been shown that the differentiation of CD4^+^ T cells into Th1 and Th2 subsets may be dependent on TCR signal strength, whereby strong TCR-induced ERK signals favor Th1 over Th2 differentiation (Yamane et al., 2005). Thus, it is possible that DGKζ controls Th1 vs. Th2 lineage commitment by regulating DAG-mediated ERK activation in T cells. Although the impact of DGKζ deficiency on Th2 responses is unknown, DGKζ KO T cells display heightened Th1-driven anti-viral and anti-tumor responses. Thus, it would be interesting to test whether the loss of DGKζ impairs protection against Th2-inducing helminth infections or beneficially promotes protection against Th2-mediated diseases such as asthma. The precise delineation of how DGKζ controls the outcome of immune responses will yield insight into how DGKζ could be targeted for the treatment of various immune-mediated disorders.

## Author contributions

All authors listed, have made substantial, direct and intellectual contribution to the work, and approved it for publication.

### Conflict of interest statement

The authors declare that the research was conducted in the absence of any commercial or financial relationships that could be construed as a potential conflict of interest.
